# 

**Published:** 1917-06-23

**Authors:** 


### CONTENTS.

PAGE

**The Churches' Opportunity : Hospital Sunday, 1917** ... ... ... ... ... ... ... ... 217

**Hospital and Institutional News** ... ... ... 219

The late Lieutenant Mackintosh—The Air Raid on London—How to Warn London Hospitals—Recuperative Hostels—Esprit de Corps at St. Bartholomew's—Another Landmark at Cardiff—A Remarkable Change at Devonport—Women on their Own—The Conditional Donation—The Abuse of the Ticket System—A Real Improvement—A Jubilee Fund still Unallotted—Meat Inspection Problems—Ulsterman's Bequests to Charities—Bequest for a New Hospital—Lotteries for Charity—The "Fifth"—State Milk—This Week's Drug Market.

**Hospital Chapels and Hospital Sunday** ... 223

**The Chapel of Guy's Hospital** ... ... ... 223

**Thomas Guy, 1645-1724** ... ... ... ... 225

**The Church's Attitude Towards Disease** ... 226

**St. Thomas's Hospital** ... ... ... ... 227

Resignation of Mr. J. G. Wainwright, the Treasurer.

**Life in a Korean Hospital** ... ... ... ... 229

A Medical Mission in the East.

PAGE

**The Matrons' and Sisters' Department** ... 231

Poor-Law Nursing in England. Poor-Law Nursing To-day.—II.

Round the Hospitals.

Continuous Growth of the College—Thousands of Nurses Join College Register—Edinburgh Badge for Royal Infirmary Nurses—The Nation's Fund for Nurses £500—More Money on its Way—Nurses'. Tribute to Bezley Thorne.

**Hospitals and their Special Needs** ... ... 233

**Nearly Two Million Sufferers Helped by our Hospitals** ... ... ... ... ... ... ... 234

A Single Year's Roll-call of the Sick.

**1916.—A Year's Work in the Hospitals and Medical Charities of London** ... ... ... 238

**The Institutional Worker** ... ... (Suppl.) 1

How to Prevent Infection in a Surgical Ward.

Answer to April Question.

Substitutes for Potatoes. Answer to May Question.

Question for June.

Enquiries and Answers.

The Sick and in Need.

War Matters.

Miscellaneous.

